# Flapping Foil-Based Propulsion and Power Generation: A Comprehensive Review

**DOI:** 10.3390/biomimetics11020086

**Published:** 2026-01-25

**Authors:** Prabal Kandel, Jiadong Wang, Jian Deng

**Affiliations:** 1 State Key Laboratory of Fluid Power and Mechatronic Systems, Department of Mechanics, Zhejiang University, Hangzhou 310027, China; zjuprabal@zju.edu.cn (P.K.);; 2 Huanjiang Laboratory, Zhejiang University, Zhuji 311816, China

**Keywords:** flapping foil, bio-inspired propulsion, power generation, flow energy harvesting, density stratification, deep reinforcement learning, unsteady aerodynamics

## Abstract

This review synthesizes the state of the art in flapping foil technology and bridges the distinct engineering domains of bio-inspired propulsion and power generation via flow energy harvesting. This review is motivated by the observation that propulsion and power-generation studies are frequently presented separately, even though they share common unsteady vortex dynamics. Accordingly, we adopt a unified unsteady-aerodynamic perspective to relate propulsion and energy-extraction regimes within a common framework and to clarify their operational duality. Within this unified framework, the feathering parameter provides a theoretical delimiter between momentum transfer and kinetic energy extraction. A critical analysis of experimental foundations demonstrates that while passive structural flexibility enhances propulsive thrust via favorable wake interactions, synchronization mismatches between deformation and peak hydrodynamic loading constrain its benefits in power generation. This review extends the analysis to complex and non-homogeneous environments and identifies that density stratification fundamentally alters the hydrodynamic performance. Specifically, resonant interactions with the natural Brunt–Väisälä frequency of the fluid shift the optimal kinematic regimes. The present study also surveys computational methodologies and highlights a paradigm shift from traditional parametric sweeps to high-fidelity three-dimensional (3D) Large-Eddy Simulations (LESs) and Deep Reinforcement Learning (DRL) to resolve finite-span vortex interconnectivities. Finally, this review outlines the critical pathways for future research. To bridge the gap between computational idealization and physical reality, the findings suggest that future systems prioritize tunable stiffness mechanisms, multi-phase environmental modeling, and artificial intelligence (AI)-driven digital twin frameworks for real-time adaptation.

## 1. Introduction

The locomotion of aquatic animals and flying insects has long inspired engineers seeking to enhance the efficiency and maneuverability of marine and aerial vehicles. Through millions of years of evolution, species such as tuna, dolphins, and birds have refined the use of oscillatory lifting surfaces, specifically wings and fins, to navigate complex fluid environments. These biological systems achieve performance metrics that often exceed conventional rotary propellers [1,2,3]. This bio-inspired mechanism, broadly termed flapping foil technology, has been divided into two distinct but physically related fields of engineering application: propulsion, where the foil imparts energy to the fluid to generate thrust, and power generation, where the foil extracts kinetic energy from the fluid flow [4]. In recent decades, research into flapping foils has expanded from fundamental fluid dynamics to applied systems for Autonomous Underwater Vehicles (AUVs) and renewable energy harvesters. The foundational principle relies on the generation of unsteady vortical structures, particularly the leading-edge vortex (LEV) and the reverse von Kármán vortex street. These structures can be manipulated to maximize thrust or energy capture depending on the operational regime [5,6,7]. Despite the simplicity of the concept, the fluid–structure interaction (FSI) governing these systems involves a complex, high-dimensional parameter space comprising kinematic, geometric, and structural variables.

### 1.1. Kinematics and Governing Parameters

To understand the dual nature of flapping foils, it is essential to define their fundamental kinematics. In a typical two-dimensional approximation, a rigid foil in time (*t*) undergoes a combined heaving translation h(t) and pitching rotation θ(t). Assuming sinusoidal motion, which is the standard baseline in theoretical and numerical studies [8,9], the motion is defined as

(1) $$h\left(t\right)=h_{0}\sin\left(2\pi ft\right)$$

(2) $$\theta \left(t\right)=\theta_{0}\sin\left(2\pi ft+\phi \right)$$

**Figure 1:**
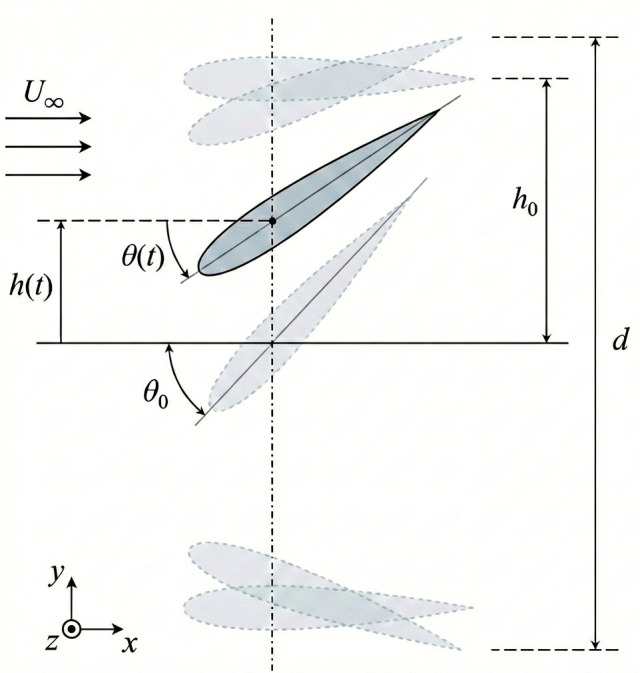
Schematic of a two-dimensional flapping foil undergoing combined heaving and pitching motion in a uniform free-stream velocity $U_{\infty }$. The schematic is shown in the *x*–*y* plane with *x* aligned with $U_{\infty }$ and *y* transverse. The sign convention is h(t)>0 in +y and θ(t)>0 for nose-up rotation about +z, measured from +x to the chord line. The solid profile indicates the instantaneous position characterized by h(t) and θ(t). The dashed profiles illustrate the motion envelope. *d* denotes the swept height used for capture-area scaling in Equation (9).

Here, $h_{0}$ is the heave amplitude, $\theta_{0}$ is the pitch amplitude, *f* is the oscillation frequency and ϕ is the phase difference between heave and pitch [3,10]. A schematic representation of these kinematic parameters and the resulting foil trajectory is shown in Figure 1.

The hydrodynamic performance of these systems is governed by nondimensional parameters that characterize flow unsteadiness. The most critical of these is the Strouhal number (St), defined as

(3) $$St=\frac{2h_{0}f}{U_{\infty }}\;\mathrm{or}\;St=\frac{fA}{U_{\infty }}$$

where *A* is the wake width (often approximated as the peak-to-peak excursion $2h_{0}$). For propulsion, optimal efficiency is typically observed within the range of 0.2<St<0.4. This range is consistent with the cruising kinematics of many swimming animals [11,12,13,14].

Two other essential parameters are the reduced frequency (*k*) and the effective angle of attack ($\alpha_{\mathrm{eff}}$). The reduced frequency serves as a measure of the degree of unsteadiness dominated by vortex shedding and is defined as

(4) $$k=\frac{\pi fc}{U_{\infty }}$$

where *c* denotes the chord length of the foil. We note that while *k* is adopted as the primary parameter throughout this review for consistency, several foundational studies cited herein utilize the alternative non-dimensional frequency notation $f^{*}=fc/U_{\infty }$. In such instances, the original $f^{*}$ values from the source material are maintained to ensure technical accuracy. The effective angle of attack determines the instantaneous loading on the foil and is critical to predict dynamic stall and LEV formation [15,16]. It is approximated as

(5) $$\alpha_{\mathrm{eff}}\left(t\right)=\arctan\left(\frac{-\dot{h}\left(t\right)}{U_{\infty }}\right)-\theta \left(t\right)$$

where $\dot{h}\left(t\right)$ represents the instantaneous heave velocity.

### 1.2. Operational Regimes and Performance Metrics

The distinction between using a flapping foil as a thruster or a turbine is mainly dictated by the relationship between the heave velocity and the hydrodynamic force. This transition is characterized by the feathering parameter (χ), defined as the ratio of the maximum pitch angle to the maximum induced angle of attack from heaving:

(6) $$\chi =\frac{\theta_{0}}{\arctan(2\pi fh_{0}/U_{\infty })}$$

As established by theory and confirmed by numerical studies, χ<1 generally results in propulsion (thrust production through the “Knoller-Betz” effect), while χ>1 results in power generation (energy extraction through negative damping or flutter) [10,16].

Hydrodynamic performance in these regimes is quantified by instantaneous power exchange P(t), defined as

(7) $$P\left(t\right)=Y\left(t\right)\dot{h}\left(t\right)+M\left(t\right)\dot{\theta }\left(t\right)$$

where Y(t) is the vertical heaving force, M(t) is the pitching moment, and $\dot{\theta }\left(t\right)$ is the instantaneous pitching velocity.

For propulsion applications, performance is measured by the time-averaged thrust coefficient (${\bar{C}}_{T}$) and propulsive efficiency ($\eta_{P}$):

(8) $${\bar{C}}_{T}=\frac{\bar{T}}{\frac{1}{2}\rho U_{\infty }^{2}c},\;\eta_{P}=\frac{\bar{T}U_{\infty }}{{\bar{P}}_{in}}$$

where ρ is the fluid density and ${\bar{P}}_{in}$ is the cycle-averaged mechanical power required to drive the foil. Let T(t) denote the instantaneous streamwise hydrodynamic force on the foil (positive in the direction of motion). The cycle-averaged thrust is $\bar{T}=\frac{1}{T_{p}}\int_{t_{0}}^{t_{0}+T_{p}}T\left(t\right)\;dt$, where $T_{p}=1/f$ and $t_{0}$ is a cycle start time selected after transients decay.

High propulsive efficiency is typically associated with the formation of a reverse von Kármán vortex street in the wake [5].

In contrast, for power generation, performance is evaluated by power extraction efficiency ($\eta_{E}$):

(9) $$\eta_{E}=\frac{{\bar{P}}_{out}}{\frac{1}{2}\rho U_{\infty }^{3}d}$$

where ${\bar{P}}_{out}$ is the time-averaged power extracted from the fluid and *d* represents the characteristic dimension of the capture area (typically the vertical sweep width of the foil motion, $d=2h_{0}+c\sin\theta_{0}$). In practice, $d\approx 2h_{0}$ is a convenient simplification only when the heave sweep dominates the capture height, so that the pitch-induced chord projection $c\sin\theta_{0}$ is small compared with $2h_{0}$.

### 1.3. Classification of Flapping Systems

The implementation of flapping foil technology varies depending on the actuation mechanism and degree of structural compliance. The current literature classifies these systems into three primary categories. The first category consists of fully active systems in which both heaving and pitching motions are kinematically prescribed by external motors. This approach is common in fundamental research [5] because it allows precise control over the Strouhal number and phase angle to map the efficiency landscape. The second category comprises semi-active (or semi-passive) systems. Typically, the heaving motion is driven (or induced by wave motion in the case of wave gliders), whereas the pitching motion is passive and controlled by a torsion spring and damper system. These systems rely on fluid–structure interactions to adjust the pitch angle, thereby reducing mechanical complexity [17,18].

The final category includes fully passive systems in which the foil is mounted on elastic supports or mechanically coupled mechanisms for both heave and pitch. Rather than relying on prescribed actuation, these systems depend on fluid–structure interaction instabilities or coupled dynamics to sustain limit-cycle oscillations. Although mechanically simple, these systems require careful tuning of structural parameters, such as mass ratios and stiffness, to initiate and maintain energy harvesting, a process governed by the principles of flow-induced instability and vortex-induced vibration [19,20]. Additionally, the introduction of chordwise or spanwise flexibility has been shown to enhance propulsive efficiency in these passive regimes [21].

### 1.4. Scope of the Review

This review aims to provide a comprehensive synthesis of the state of the art in flapping foil technology, bridging the gap between propulsion and power generation. Building upon foundational reviews that have provided in-depth analyses of these domains individually [3,10,22], we attempt to unify these domains by focusing on the shared unsteady aerodynamic framework that governs both operational modes. The remainder of this paper is organized as follows: Section 2 outlines the foundational theoretical frameworks; Section 3 surveys the experimental investigations; and Section 4 details the numerical studies, highlighting the role of computational fluid dynamics (CFD) in exploring high-dimensional parameter spaces. Section 5 discusses the current technical challenges, limitations, and future outlook of this field. Finally, Section 6 presents the conclusions.

## 2. Theoretical Frameworks

The fundamental understanding of flapping foil aerodynamics and hydrodynamics is rooted in a rich history of theoretical and analytical work. Although these studies are often based on simplifying assumptions, they provide invaluable physical insights into the mechanisms of propulsion and power generation. This section reviews the key theoretical frameworks, from foundational potential flow theories to modern analyses that incorporate flexibility and complex interactions.

### 2.1. Theoretical Studies on Flapping Foil-Based Propulsion

#### 2.1.1. Foundational Linear Potential Flow and Modern Vortex Theories

The earliest theoretical explanations for thrust generation from an oscillating airfoil are commonly attributed to the Knoller–Betz effect, first described independently by Knoller [23] and Betz [24]. This principle states that an oscillating foil creates an effective angle of attack relative to the flow direction. For example, in an upward-moving flow (or downward foil stroke), the resultant aerodynamic force vector tilts forward, generating a thrust component [9,25]. Following this concept, the seminal works of Theodorsen [26], Garrick [8], and von Karman and Sears [27] established a linear potential flow theory for an oscillating airfoil. This theoretical framework is based on the assumptions of inviscid, incompressible flow, small-amplitude motions, and an approximately flat, nondeforming vortex wake [28,29]. The theory by Garrick [8] predicts that thrust arises from two primary contributions: the leading-edge suction force and the projection of the pressure force on the airfoil in the direction of flight [29]. An important result of this theory is that whereas plunging airfoils can generate thrust across all frequencies, pitching foils only produce thrust above a critical frequency that depends on the pivot location [30].

While foundational, the theory by Garrick [8] has limitations. Fernandez-Feria [31] revisited the linearized theory using vortical impulse theory, which accounts for the complete vorticity distribution on both the airfoil and its wake. This approach corrects the thrust prediction of the classical model by consistently including the effect of wake vorticity, and provides better agreement with numerical and experimental data over a wider range of Reynolds and Strouhal numbers than the original Garrick formulation [29,31].

#### 2.1.2. Theories for Fish-like Swimming

Although these theories were first developed to explain the wave-like swimming of fish, they also help to describe how oscillatory motions generate thrust in modern flapping foil propulsion. Several theoretical models have been established for the undulatory motion characteristics of fish swimming. Lighthill [32] proposed the large-amplitude elongated-body theory and Wu [33] developed the two-dimensional waving-plate theory. Together, these works form foundational contributions in this area. Lighthill’s work provides a detailed framework for how slender fish generate thrust and lateral forces through body undulations, whereas Wu’s theory models a flexible two-dimensional plate in high-Reynolds-number flow and has been widely used as an idealized description of high-aspect-ratio propulsive fins. This framework enables the calculation of thrust, power, and efficiency, and allows the analysis of their dependence on kinematic parameters, such as wavelength, phase speed, and amplitude distribution [33]. Subsequent experimental and theoretical studies, particularly those of Triantafyllou et al. [11], have shown that efficient propulsion is associated with the formation of a reverse von Kármán vortex street in the wake of the fish. This wake corresponds to a jet-like flow, indicating a net transfer of momentum to the fluid that produces thrust.

#### 2.1.3. Theories for Flexible and Tandem Foils

**Figure 2:**
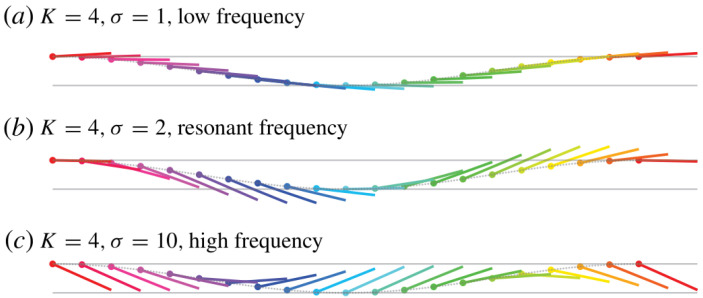
Emergent passive-pitching kinematics of a heaving-driven flexible wing from Moore [34]. The wing is shown at three representative reduced frequencies: (**a**) low frequency produces only slight pitching; (**b**) near resonance the pitching becomes pronounced (“flinging”), increasing the trailing-edge amplitude; (**c**) at higher frequency the pitching becomes out-of-phase, reducing trailing-edge motion. Notation: Moore [34] denotes reduced frequency by $\sigma =\pi fc/U_{\infty }$, which is equivalent to *k* in Equation (4); their stiffness parameter *K* (spring stiffness relative to fluid loading) is *not* the same as the waveform-shaping parameter *K* (e.g., $K_{\theta }$, $K_{h}$) used later for nonsinusoidal motions. Reprinted with permission from Moore [34].

Recognizing that natural propulsors are often flexible, theoretical studies have been expanded to account for foil deformation. Moore [34] provided analytical insights into how flexibility influences flapping propulsion using a passive pitching model. Their results demonstrated that the resonance between the driving frequency and the natural frequency of the foil can significantly enhance the thrust. As illustrated in Figure 2, this enhancement is associated with a pronounced near-resonant passive pitching response (often described as a “flinging” motion), which increases trailing-edge excursion relative to off-resonant conditions.

However, their inviscid linear model did not capture the corresponding peak in propulsive efficiency at resonance, suggesting that viscous effects may be required to explain the efficiency optima observed in experiments. Building on this, Alaminos-Quesada and Fernandez-Feria [35] developed general analytical expressions for the aerodynamic forces and moments acting on a flexible foil undergoing prescribed undulatory motion using vortical impulse theory in the linear potential limit. Their formulation allows the identification of kinematic conditions, including reduced frequency, deflection amplitude, and phase shift, which optimize thrust generation and propulsion efficiency.

### 2.2. Theoretical Studies on Flapping Foil-Based Power Generation

#### 2.2.1. Foundational Concepts and Transition Criteria

The concept of using a flapping foil for energy harvesting was first theoretically proposed by Wu [36], who demonstrated that an oscillating foil could extract energy from an unsteady current, such as surface waves. Subsequently, McKinney and DeLaurier [4] introduced the “wingmill” concept, establishing that energy extraction is also feasible from a uniform flow [10]. As described in Section 1, the operational mode of the foil is dictated by the feathering parameter χ (Equation (6)). Although propulsion generally occurs when χ<1, the system transitions to the power-extraction regime when χ>1 [10]. In the latter regime, the effective angle of attack becomes negative as the foil passes through its mean position, aligning the lift force with the heaving motion, and allowing the fluid to perform work on the foil [16]. Near χ≈1, the foil operates in a feathering mode, producing minimal net thrust or power [10]. This criterion remains the fundamental guideline for selecting flapping foil kinematics for energy harvesting applications.

#### 2.2.2. Theoretical Models for Passive and Semi-Passive Systems

Theoretical modeling of flapping foils differs significantly depending on whether the system is semi-passive, requiring external actuation, or fully passive, relying on aeroelastic instability. For semi-passive systems, the theoretical challenge is to model the coupling between a prescribed motion (e.g., pitching) and an induced energy-extracting response (e.g., heaving). To capture nonlinear viscous effects, such as the formation of the LEV, which are overlooked by simpler thin-plate approximations, Zhu and Peng [37] employed a Navier-Stokes model. Their analysis showed that net positive power extraction is possible when the energy extracted from the induced heaving motion exceeds the power required for active pitching. In contrast, models for fully passive systems focus on predicting the onset of flow-induced flutters. The primary theoretical tool is linear stability analysis, which is used to determine the critical flow velocity or flutter speed at which self-sustained oscillations emerge. Both Peng and Zhu [19] and Fernandez-Feria and Sanmiguel-Rojas [38] used this framework to identify parametric regimes in which stable periodic motions suitable for energy harvesting can occur. Although this analytical approach can predict the onset of instability, it cannot describe the subsequent nonlinear finite-amplitude oscillations. Therefore, these studies complemented their stability analysis with numerical simulations to quantify the actual power output (which cannot be predicted by linear theory) and investigate the complex fluid dynamics, such as flow separation and vortex shedding, that govern the system’s performance in its fully developed, large-amplitude oscillatory state.

#### 2.2.3. Wave-Devouring Propulsion (WDP)

A specialized area of theoretical study is Wave-Devouring Propulsion (WDP), where hydrofoils extract energy from incident waves to generate thrust. Building on his earlier contributions to unsteady airfoil theory, Wu [36] established the theoretical basis for energy extraction by a wing oscillating in waves, demonstrating that an oscillatory hydrofoil can absorb energy from the incident wave field, provided that the basic flow contains a wave component. However, this early formulation assumed that the hydrofoil was sufficiently far from the free surface to ignore the boundary effects. To address this limitation, Isshiki [39] developed a two-dimensional model that extended Wu’s theory by incorporating free-surface effects approximately and applied it to assess the feasibility of wave-devouring propulsion for a non-oscillating hydrofoil (Linear Wells Turbine). This line of research was subsequently extended by Isshiki and Murakami [40], who clarified the fundamental aspects of passive-type WDP. Although their experiments confirmed thrust generation, their theoretical analysis revealed that introducing wave-making damping terms into the equations of motion was necessary to correct the theoretical predictions at low advance speeds, where resonance occurs. Subsequent theoretical models, such as that of Grue [41], further developed frequency-domain integral equation approaches for analyzing foils near a free surface. This study confirmed that free surface effects strongly influence the vortex wake and forces on the hydrofoil; however, significant wave energy extraction (up to 75%) remains possible in both the head and the following seas. Finally, regarding efficiency limits, Young et al. [10] noted that while the Betz limit of 59.3% is the accepted theoretical maximum for rotary turbines, its direct applicability to flapping foils remains a subject of research.

In summary, theoretical models provide the core physical interpretation of how prescribed kinematics, phase relationships, and fluid–structure coupling govern thrust generation and power extraction. They also clarify regime transitions through compact criteria such as feathering and stability thresholds, while highlighting where viscous, nonlinear, and free-surface effects limit simplified formulations. These insights motivate the experimental studies that follow, which test these mechanisms under controlled conditions and quantify performance in realizable systems.

## 3. Experimental Studies

Experimental investigations have been fundamental for understanding the complex fluid–structure interactions that govern the performance of flapping foil systems. Although numerical simulations provide detailed insight into flow physics, physical experiments serve as the definitive validation for theoretical models and offer tangible proof of concept for real-world applications. This section reviews key experimental works and categorizes studies based on their primary objectives: propulsion or power generation.

### 3.1. Propulsion-Based Experimental Studies

The study of flapping foil propulsion has been heavily inspired by the efficient locomotion of aquatic animals. Experimental research has been crucial for translating these biological principles into engineered systems, focusing on optimizing thrust and efficiency by exploring a wide range of kinematic, geometric, and material parameters.

#### 3.1.1. Influence of Kinematic Parameters

**Figure 3:**
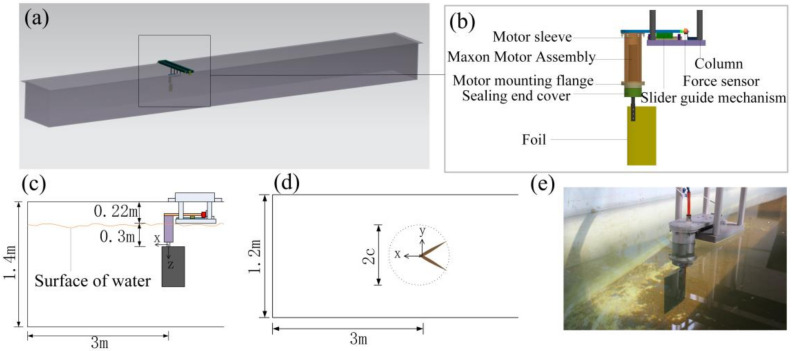
Layout of experimental platform from Ding et al. [44]. The setup includes: (**a**) the overall platform model; (**b**) the detailed device structure highlighting the guide rail slider mechanism used to decouple forces; (**c**,**d**) installation diagrams with coordinates; and (**e**) a physical photograph of the device in the water channel. Reprinted from Ding et al. [44].

A foundational experimental study by Anderson et al. [5], conducted in a towing tank at a Reynolds number Re = 40,000 (defined as $Re=U_{\infty }c/\nu$, where ν is the kinematic viscosity of the working fluid), combined force measurements and Digital Particle Image Velocimetry (DPIV) to map the performance of a harmonically oscillating NACA 0012 hydrofoil. They demonstrated that a high propulsive efficiency, reaching up to 87%, was achieved under specific conditions associated with the formation of a reverse von Kármán vortex street. The optimal parameters were found to be a Strouhal number St between 0.25 and 0.4, large heave-to-chord ratio, large maximum angle of attack ($15^{{}^{\circ}}$–$25^{{}^{\circ}}$) and pitch-heave phase angle of approximately $75^{{}^{\circ}}$. Following this, Schouveiler et al. [42] conducted a systematic investigation into the effects of the Strouhal number and the maximum angle of attack, confirming that optimal parameter combinations could yield peak efficiencies of over 70%. Other studies have focused on simpler kinematics such as pure plunge (heave). Joseph C. S. Lai and Max F. Platzer [43], using a water tunnel and Laser Doppler Velocimetry (LDV) over a Re range of 500–21,000, studied a plunging NACA 0012 hydrofoil. They identified the nondimensional plunge velocity (the ratio of the maximum plunge velocity to the freestream speed) as the critical parameter governing the transition from a drag-producing wake to a thrust-producing jet. More recently, Ding et al. [44] constructed a novel experimental apparatus to scrutinize frequency-amplitude parameters. As shown in Figure 3, this custom-built platform utilizes a guide rail slider mechanism to physically decouple thrust, lift, and torque, ensuring that only the force in the direction of the slider movement is measured. The corresponding force-measurement subsystem in the original setup consists of a force sensor and a data-acquisition chain, which acts as the measurement “receiver” for the decoupled loads.

Using this setup, they formulated a response surface connecting the propulsive efficiency to these motion parameters and experimentally determined the highest efficiency for their setup at a frequency of 2 Hz and a flapping amplitude of $40^{{}^{\circ}}$. These studies collectively highlight that the precise tuning of kinematic parameters, often correlated through the Strouhal number, is essential for achieving high-performance propulsion.

#### 3.1.2. Effect of Geometric Parameters and Foil Morphology

Beyond the kinematics, the physical shape and configuration of the foil significantly affect its hydrodynamic performance. The key geometric parameters investigated experimentally include the aspect ratio (AR=b/c, where *b* is the span) and location of the pitching axis (pivot point). Ding et al. [44] examined the effect of AR by varying both the span and chord length of the hydrofoil. Their experiments showed that when the chord length was constant, increasing the span length led to a gradual improvement in propulsive efficiency. Conversely, when the span was held constant, increasing the chord length caused the efficiency to follow a parabolic trend, first increasing and then decreasing. Ayancik et al. [45] also explored the role of AR through a combination of inviscid simulations and fixed-velocity experiments in a water channel at a Re of 30,000, which were used to validate a set of 3D scaling laws for pitching propulsors. Their work confirmed that accounting for the AR is critical for accurately predicting the thrust and power.

**Figure 4:**
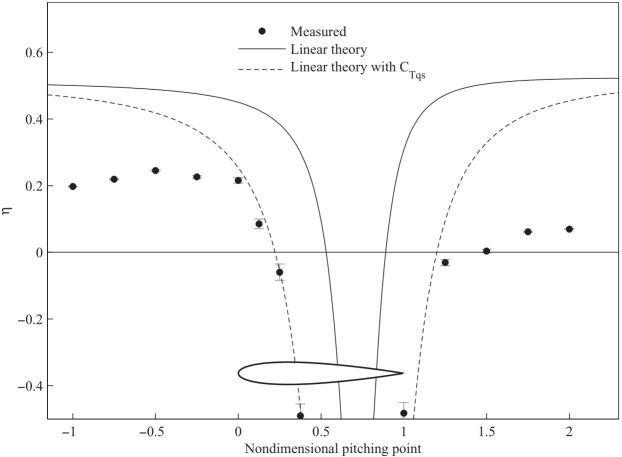
Propulsive efficiency (η, denoted $\eta_{P}$ in this review) plotted against the nondimensional pitching point (p/c, where *p* is the distance from the leading edge to the pitch axis measured along the chord) for reduced frequency k=2 and amplitude $\theta_{0}=8^{{}^{\circ}}$. The experimental data (dots) reveal an optimal pivot location ahead of the leading edge. The solid line represents standard inviscid linear theory. The dashed line represents linear theory corrected with a quasistatic drag coefficient ($C_{Tqs}$), which accounts for viscous drag averaged over the oscillation cycle. Reprinted with permission from Mackowski and Williamson [46].

The location of the pivot point for the pitching foils is another critical design choice. Classical linear theory suggests that propulsive efficiency increases as the pivot point moves further away from the airfoil [8]. However, Mackowski and Williamson [46] conducted experiments that challenged this, demonstrating that an optimal pivot location exists near the hydrofoil that maximizes the propulsive efficiency. As shown in Figure 4, their measurements reveal a clear efficiency peak when the pitching axis is located approximately a half-chord length ahead of the leading edge (nondimensional pitching point ≈−0.5), a trend not captured by linear theory alone.

#### 3.1.3. Role of Foil Flexibility and Passive Dynamics

In a low Reynolds number (Re≈1035) hydrodynamic tunnel, Marais et al. [47] used Particle Image Velocimetry (PIV) to compare a rigid foil with a flexible hydrofoil of the same geometry, finding that flexibility had a significant impact on both thrust and wake stability. The momentum balance from their experiments showed that the average thrust of the flexible hydrofoil was up to three times greater than that of the rigid foil. This was attributed to the faster vortex formation and enhanced flow around the trailing edge owing to the deformation of the hydrofoil. Furthermore, flexibility was found to inhibit symmetry breaking of the propulsive jet, leading to a more stable wake. Similarly, Sharma and Dutta [48] studied the effect of chordwise flexibility on the drag-thrust transition of a NACA 0015 airfoil in a subsonic wind tunnel over a Re range of 1200–5100. Their experiments revealed that the flexible airfoil achieved approximately twice the net thrust of its rigid counterpart, with the minimum drag coefficient ratio decreasing from −1.55 for the rigid foil to −3.17 for the flexible airfoil. An alternative approach for incorporating passive dynamics involves the use of springs. Mackowski and Williamson [46] innovatively used a “Cyber-Physical” system to attach virtual passive springs to a pitching airfoil in the heave (transverse) direction. This allows the single-actuator system to combine pitching and heaving motions, boosting the maximum propulsive efficiency from 25% (pitching only) to 35% (pitching with passive heave). Taking this concept further, Wang et al. [49] investigated a fully passive NACA 0024 hydrofoil for marine propulsion in a wave tank, where both heave and pitch motions were driven by waves and controlled using springs. Their experiments explored how varying the heave and pitch spring stiffness affects the generated thrust, identified an optimal pitch stiffness, and noted that the heave spring contributed to a harmonic force response.

#### 3.1.4. Operational Environment Effects

**Figure 5:**
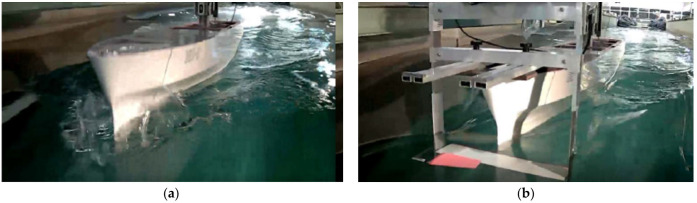
Photographs of the experimental tank tests for the ferry hull model in head waves. Panel (**a**) shows the baseline tests without the dynamic wing, while panel (**b**) shows the model with the active flapping thruster installed at the bow. Reprinted with permission from Belibassakis et al. [51].

The performance of a flapping foil can also be influenced by its interaction with the surrounding environment and integration into a larger vehicle. Experimental studies have explored phenomena such as the ground effect and the application of foils to augment ship propulsion. Quinn et al. [50] conducted experiments in a water channel at Re=4700 on a pitching hydrofoil operating near a solid boundary (ground effect). Their results showed that proximity to the ground significantly enhanced propulsive performance. The time-averaged thrust increased monotonically as the foil approached the boundary, increasing by approximately 40% at the optimal distance with little to no extra power cost. The experiments also revealed the formation of a stable equilibrium point at which the net lift on the foil was zero. At a larger scale, experiments have been conducted on flapping foils integrated with ship models. Belibassakis et al. [51] tested a ferry model in a towing tank with an actively controlled NACA 0012 dynamic wing mounted at the bow. The goal was to harness energy from the ship’s vertical motions in waves. As shown in Figure 5, tests were conducted in head waves to compare the hull’s performance with and without the active thruster. The experiments demonstrated that the foil acts as a damper: its operation significantly reduced the ship’s heaving and pitching amplitudes. This stabilization effect, combined with the thrust generated by the foil, resulted in an overall performance enhancement (i.e., a reduction in total resistance) of 15–30% in head waves around the ship’s resonance frequency.

For a more direct application, Mannam and Krishnankutty [52] performed model tests on a remotely operated surface ship fitted with an oscillating caudal fin. In the self-propulsion mode, a maximum thrust of 8.6 N was recorded, whereas in the bollard pull condition, the thrust reached 14.8 N, demonstrating the practical viability of flapping foils for vehicle propulsion.

### 3.2. Power Generation Based Experimental Studies

In addition to propulsion, oscillating foils can be configured to operate in reverse, extracting kinetic energy from fluid flow to generate power. These systems, often called oscillating foil energy converters (OFECs), have been investigated experimentally as potential alternatives to conventional rotary turbines for harvesting wind and hydrokinetic energy.

#### 3.2.1. Influence of Kinematic and Operational Parameters

Similar to propulsion, the performance of the OFEC is highly dependent on its operating parameters. An early experimental investigation of this concept was conducted by McKinney and DeLaurier [4] on an oscillating-wing windmill they termed the “Wingmill.” Their working model, tested in a wind tunnel, was shown to be capable of efficiencies comparable to rotary designs, with a maximum measured efficiency of 28.3% for a configuration with a pitching amplitude of $30^{{}^{\circ}}$. Expanding on the fundamental kinematic requirements in hydrodynamic environments, Simpson et al. [53] demonstrated that hydrofoils performing simple sinusoidal sway and yaw motions can efficiently extract energy from unsteady flows. In their water tank experiments using NACA 0012 hydrofoils, they achieved overall efficiencies of up to 43±3%. Their study highlighted the critical role of hydrofoil geometry, showing that efficiency is closely related to aspect ratio; while the highest AR of 7.9 achieved 43% efficiency at a Strouhal number of 0.4, efficiency dropped to 29% and 17% for aspect ratios of 5.9 and 4.1, respectively.

Recent studies have focused on optimizing semi-active or active systems. Jiacheng et al. [54] developed a new semi-active flapping airfoil power generator (FAPG) and used an orthogonal experiment method in a fan-driven open-flow test setup to systematically explore the influence of pitching amplitude, pitching axis position, and pitching period. Their results across multiple wind speeds showed that the pitching period had the greatest impact on the mean net output power, as shorter periods significantly increased the power consumed by the pitching motion itself, leading to a reduced net energy harvesting performance. Totpal et al. [55] experimentally investigated an OFEC operating at low reduced frequencies (k=0.04–0.08), corresponding to off-peak conditions with high flow velocities. Their results revealed that at the upper end of this range (k=0.08), the conditions for optimal efficiency (pitching amplitude $\theta_{0}=45^{{}^{\circ}}$ and phase shift $\phi =90^{{}^{\circ}}$) differ significantly from those for optimal power extraction ($\theta_{0}=60^{{}^{\circ}}$, $\phi =60^{{}^{\circ}}$). Although this highlights a crucial trade-off between maximizing conversion efficiency and absolute power output, this distinct optimization sensitivity decreases as *k* decreases to 0.04, where performance becomes relatively insensitive to variations in $\theta_{0}$ and ϕ due to premature LEV detachment.

#### 3.2.2. Effect of Foil Deformation and Flexibility

The potential of foil flexibility to enhance performance has also been explored in the context of energy harvesting. Although flexibility has shown consistent benefits for propulsion, its role in power generation appears more nuanced and highly dependent on the dynamics of the system. Totpal et al. [55] conducted a unique experimental study on an OFEC with an inertia-induced, passively deforming leading edge. They found that, while the flexible leading edge could enhance the aerodynamic lift force during certain parts of the oscillation cycle, this enhancement occurred early in the cycle at lower heaving velocities. The timing of the deformation, which was governed by the inertia of the foil, was not synchronized with the peak power-producing phase of the motion. Consequently, passive flexibility did not lead to an overall improvement in power output and, in some cases, reduced performance. This result indicates that for flexibility to be beneficial in energy harvesting, the deformation must be carefully tuned to the foil kinematics, a task that may require active control or more sophisticated passive designs. In contrast to leading-edge deformation, Siala and Liburdy [56] investigated the capabilities of an oscillating wing with a passively actuated trailing edge. Their setup utilized a “forward pitching” configuration, where the pitching axis was located well behind the wing center of mass, coupled with a trailing edge connected via a torsion rod. They found that decreasing the natural frequency of the trailing edge (increasing flexibility) closer to the imposed oscillation frequency increased the magnitude of the lift and moment forces, thereby increasing the mean power output. Unlike the leading-edge findings, the passive trailing edge response increased the effective angle of attack in a manner strongly correlated with improved energy harvesting potential.

In summary, experimental studies establish reliable performance trends and validate the unsteady mechanisms that enable efficient propulsion and energy harvesting. They also highlight practical sensitivities to kinematics, flexibility, and facility constraints that are not fully captured by idealized theory. These limitations motivate numerical studies, which can isolate individual effects and explore broader parameter spaces than are feasible in laboratory testing.

## 4. Numerical Studies

### 4.1. Numerical Studies on Flapping Foil-Based Propulsion

Numerical studies on flapping foil propulsion are primarily driven by the goal of understanding and optimizing the generation of thrust and propulsive efficiency for applications in bio-inspired underwater and aerial vehicles. These computational studies allow for the systematic exploration of a wide design space that is often difficult to investigate experimentally. In most CFD studies, thrust is obtained from the streamwise component of the integrated pressure and viscous forces on the foil surface and then cycle-averaged over one period to compute $\bar{T}$ and ${\bar{C}}_{T}$ through Equation (8). The mechanical power input is computed from the force–velocity and moment–rotation terms (Equation (7)) and averaged to obtain ${\bar{P}}_{in}$, from which the propulsive efficiency is evaluated as $\eta_{P}=\bar{T}U_{\infty }/{\bar{P}}_{in}$ (Equation (8)). The following sections delve into the key areas of this research, beginning with the fundamental influence of the foil shape and prescribed motion, followed by investigations into advanced biomimetic trajectories, the critical role of structural flexibility and fluid–structure interaction (FSI), the hydrodynamic benefits of multi-foil configurations, and finally, the performance of these systems in complex, real-world flow environments.

#### 4.1.1. Influence of Kinematic and Geometric Parameters

The performance of a flapping foil is highly sensitive to its shape and motion characteristics. The fundamental numerical work of Guglielmini and Blondeaux [57] employed a two-dimensional (2D) Navier–Stokes solver based on a stream-function vorticity formulation to analyze the propulsive efficiency of oscillating foils, laying the foundation for more complex viscous flow simulations. Building on this fundamental understanding, Isogai et al. [58] used Navier-Stokes computations for a NACA 0012 foil at Re = 100,000 to investigate the effect of dynamic stall. By varying the reduced frequency (up to k=1.0 based on semi-chord) and phase difference, they determined that although unsteady motion can enhance lift, dynamic stall plays a significant role in performance degradation when kinematic parameters deviate from the optimal conditions (typically around k=0.5 and $\phi =90^{{}^{\circ}}$). Subsequent studies have focused on optimizing kinematic parameters to maximize thrust while mitigating such degradation. Joda et al. [59] simulated a NACA 0012 airfoil at Re = 10,000 for Micro Aerial Vehicle (MAV) applications using an incompressible CFD solver, prescribing a heaving amplitude of one chord length and pitching amplitudes varying up to $45^{{}^{\circ}}$. They observed that thrust increased with a reduced frequency, whereas propulsive efficiency peaked at a reduced frequency of 0.28 before declining. Wang et al. [60] conducted unsteady Reynolds-averaged Navier–Stokes (URANS) simulations at higher Reynolds numbers (Re up to $2.4\times 10^{5}$). Investigating reduced frequencies (*k*) up to 10.05 and oscillating amplitudes up to $24^{{}^{\circ}}$, they highlighted that 3D effects, such as lateral vortices, could reduce the mean thrust by more than 13% compared to 2D predictions.

**Figure 6:**
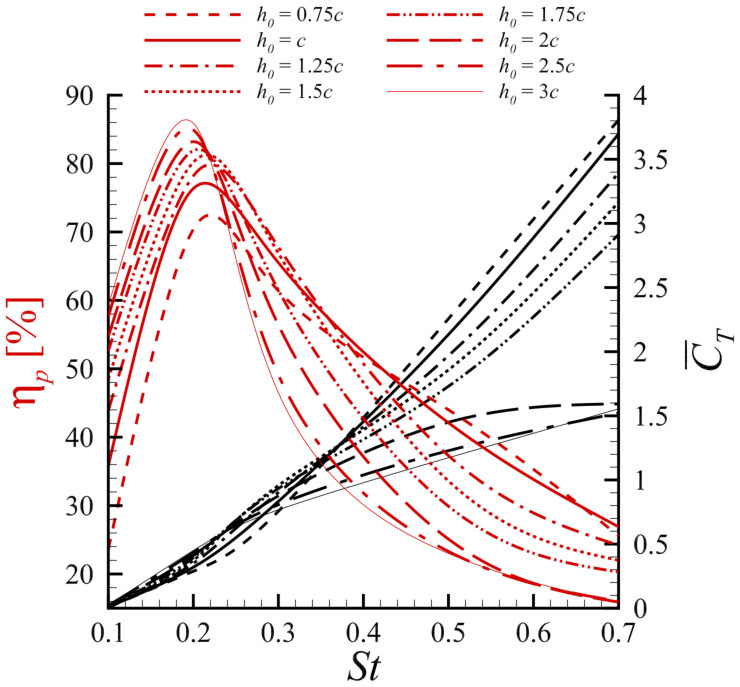
The dependence of propulsive efficiency ($\eta_{p}$, left axis; corresponding to $\eta_{P}$ in this review) and mean thrust coefficient (${\bar{C}}_{T}$, right axis) on the Strouhal number (St) for varying heave amplitudes ($h_{0}$). The data are shown for a representative kinematic configuration with a fixed pitch amplitude $\theta_{0}=15^{{}^{\circ}}$ and phase angle $\phi =90^{{}^{\circ}}$. The results indicate a global efficiency optimum in the range 0.19<St<0.3. Reprinted from Alberti et al. [61].

Synthesizing these kinematic factors, Alberti et al. [61] used a high-order discontinuous Galerkin (DG) solver with the Spalart–Allmaras RANS model to study a NACA 0015 hydrofoil at Re = 300,000. As illustrated in Figure 6, they observed that while the mean thrust coefficient increased monotonically with the Strouhal number (St), the propulsive efficiency exhibited a distinct peak in the range 0.2<St<0.3. Furthermore, they found that at higher Strouhal numbers, lower heave amplitudes were required to maintain efficiency by avoiding the onset of deep stall vortices. Other numerical methods have yielded similar optimization insights; Ren et al. [62] utilized a discrete vortex method (DVM) at Re = 40,000, finding that an optimal propulsion efficiency was achieved with a heaving amplitude of 1.25c and a pivot location of 0.45c. Beyond kinematics, the cross-sectional geometry of the foil significantly affects the propulsive efficiency. Ashraf et al. [63] used a 2D Navier-Stokes solver to investigate NACA airfoils across a wide range of Reynolds numbers (Re=200–$2\times 10^{6}$). They found that at low Re, thin airfoils outperformed thick airfoils, whereas at higher Re, thicker leading edges resulted in higher thrust and efficiency values. Kelly et al. [64] further challenged the common practice of using standard NACA profiles by employing an immersed boundary method to analyze shapes generated via class-shape transformation (CST). Their findings revealed that the propulsive efficiency of the foil is highly sensitive to its shape, particularly the thickness between 18% and 50% of the chord length.

Finally, the 3D planform shape is critical in this study. Dong et al. [65] (Re = 100–400) and Shao et al. [66] (Re=200) both used immersed boundary methods to study the effect of AR at low Re. Dong et al. [65] found that for thin ellipsoidal foils, thrust and efficiency gains were significant when increasing AR from 1.27 to 2.55, but less significant when increasing further to 5.09. Moving toward biomimetic planforms, D and Krishnankutty [67] used STAR-CCM+ to analyze a crescent-shaped (lunate) hydrofoil with a NACA 0012 section at 4 knots (approx. Re = $3\times 10^{5}$). They mimicked fish tail motion using a dual-hinge actuation system and found optimal efficiency at a phase difference of $150^{{}^{\circ}}$.

#### 4.1.2. Advanced Motion Trajectories and Multi-Foil Configurations

**Figure 7:**
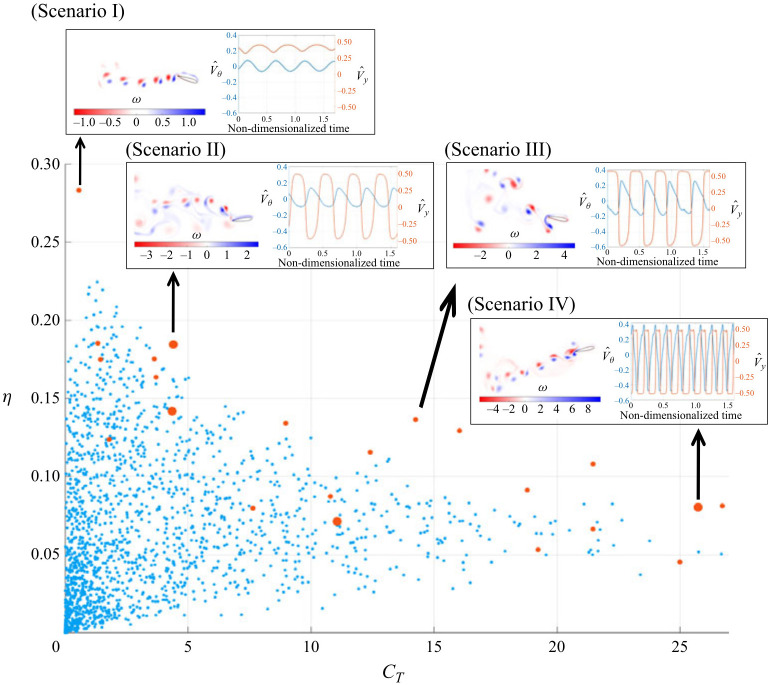
Hydrodynamic performance ($C_{T}$ vs. η, where η corresponds to $\eta_{P}$ in this review; here $C_{T}$ denotes the cycle-averaged thrust coefficient ${\bar{C}}_{T}$ as defined in Equation (8)) of sinusoidal motions (blue dots) vs. deep reinforcement learning (DRL)-optimized trajectories (red dots). The blue dots form a Pareto frontier representing the limit of sinusoidal performance. Red dots (Transformer-based proximal policy optimization, PPO) break this frontier, achieving superior thrust and efficiency. Insets display non-parametric velocity profiles (${\hat{V}}_{\theta }$ and ${\hat{V}}_{y}$ denote normalized pitch-rate and heave-velocity signals) and the resulting wake vorticity. Reprinted with permission from Wang et al. [74].

Beyond simple sinusoidal motion, researchers have numerically explored advanced trajectories to enhance propulsion. Inspired by experimental findings that the explicit control of the angle of attack can improve performance [15], numerical studies have implemented complex kinematics. Tuncer and Kaya [68] employed a gradient-based optimization algorithm coupled with a Navier–Stokes solver at Re=10,000 and a reduced frequency k=1.0 to maximize thrust and propulsive efficiency by optimizing pitch and plunge amplitudes and their phase shift. More biomimetic trajectories have been studied. Azad et al. [69] and Swain et al. [70] investigated fishtail-like motions. Using a dynamic mesh arbitrary Lagrangian–Eulerian (ALE) framework, Azad et al. [69] compared elliptical and fishtailed trajectories for single and tandem NACA 0012 foils at Re=1173 with a heaving amplitude of $h_{0}/c=0.5$, finding that elliptical flapping produced a stronger reverse von Kármán vortex street and higher thrust. Swain et al. [70] used ANSYS Fluent to focus on a fishtailed trajectory for tandem foils, demonstrating that this motion, combined with an optimal inter-foil spacing of 2–3 chord lengths, could enhance the downstream foil’s efficiency by up to 41%. Zhou et al. [71] used the commercial solver FINE/Marine to conduct a 3D fluid–structure interaction (FSI) analysis of a semi-active flapping foil driven by a swing arm (where *L* is the swing-arm length) with arm length ratios L/c of 3.0, 5.0, and 7.5 at a Re up to $9\times 10^{5}$, which naturally follows a circular arc trajectory. They found that increasing the swing arm length improved the peak efficiency. Neogi et al. [72] used a stabilized finite element moving mesh formulation at Re=1100 and a reduced frequency $f^{*}=0.2$ (defined in the source as $f^{*}=fc/U_{\infty }$, so that $k=\pi f^{*}$ in Equation (4)) to study a NACA 0015 foil with combined heaving and a prescribed trailing-edge morphing, analyzing how the morphing position and amplitude influence propulsion. They determined that propulsion is governed by the interplay between a thrust-generating LEV and a drag-inducing trailing-edge vortex (TEV), and found that optimal performance is achieved at low morph positions and intermediate morph amplitudes, which creates an ideal balance between these two competing effects. A novel approach for motion optimization was introduced by Bao et al. [73], who coupled a deep reinforcement learning (DRL) algorithm with CFD simulations performed using the open-source Lilypad solver. This method enabled an intelligent agent to autonomously discover optimal flapping trajectories for a hydrofoil to maximize its thrust generation. The study demonstrated that the DRL agent could not only identify the best parameters within a conventional set of sinusoidal motions but also outperform these human-designed strategies. This was most evident in non-parametric searches, where the agent, given the freedom to define its own motion, developed novel and more complex flapping patterns that generated superior thrust compared to the best results from an exhaustive brute-force parametric search. Building on the potential of DRL for non-parametric path planning, Wang et al. [74] proposed a training framework based on the Proximal Policy Optimization (PPO) algorithm and a Transformer architecture to optimize hydrofoil trajectories at Re=1173. To address the challenge of vast exploration spaces in non-parametric control, they initialized the policy using expert demonstrations from sinusoidal motions on the Pareto frontier. As illustrated in Figure 7, their results demonstrated that the DRL-optimized trajectories could significantly outperform sinusoidal counterparts in both thrust and efficiency. Through flow visualization (see insets in Figure 7), they identified that the agent learned to adaptively adjust the phases between the foil motion and the shedding vortices, thereby manipulating the wake morphology to enhance hydrodynamic performance.

Aerodynamic interactions in multi-foil systems, such as those in dragonfly flight or fish schools, have been modeled numerically to understand performance augmentation. Tuncer and Platzer [75] used a multiblock Navier-Stokes solver at a high Reynolds number of $Re=3.0\times 10^{6}$ to study a tandem configuration with a flapping leading airfoil and a stationary trailing airfoil, examining the gust response of the trailing foil. As previously mentioned, the works of Swain et al. [70] and Azad et al. [69] explored tandem foils with specialized trajectories, highlighting the importance of inter-foil spacing and phase angle in constructive wake interaction. Extending this further, Raut et al. [76] used direct numerical simulations (DNS) at Re = 10,000 to guide the design of multifoil wave-assisted propulsor (WAP) systems. They found that an optimal arrangement of two NACA 0015 foils could increase the trailing foil thrust by up to 80%, and they proposed three- and four-foil configurations that increased the total thrust by 49% and 67%, respectively, compared to isolated foils.

#### 4.1.3. Fluid–Structure Interaction and Flexible Foils

The passive deformation of foils is crucial for natural propulsion. Numerical FSI studies have provided insights into these effects. Mysa and Venkatraman [77] conducted numerical experiments on a 1D elastic solid in a 2D viscous fluid at Re=1000 to establish the role of foil deflection topology in vorticity generation. Shi et al. [78] employed a fully coupled FSI solver to investigate a foil with time-varying flexibility at Re=1000 and a Strouhal number of 0.5, demonstrating that periodic stiffness changes could enhance thrust by up to approximately 52%, while the highest propulsion efficiency remained almost the same, by optimizing the phase between flexibility variation and heaving motion. Qi et al. [17] used URANS simulations in Fluent to study a semi-active flapping foil (prescribed heave, passive pitch) with a NACA 0012 profile at Re = 42,000, with a baseline heaving amplitude $y_{0}=2c$. They analyzed the influence of the mass ratio and damping coefficient, finding that while the effect of the mass ratio depended on the reduced frequency, the damping coefficient had a severely adverse effect on both the output power and efficiency. Raut et al. [79] used a sharp-interface immersed boundary method to model a wave-induced propulsor at Re=10,000, where heaving of a NACA0015 foil ($h_{0}/c=0.5$) is prescribed and pitching is passive. They employed `energy maps’ and a force-partitioning method to understand the FSI and develop a phenomenological model for thrust generation. The force-partitioning method revealed that the thrust generation was dominated by the LEV. Their parametric study also showed that the performance was optimized when the pitch axis was located near the leading edge of the foil, as locations further downstream could result in chaotic oscillations or significantly diminished thrust.

#### 4.1.4. Propulsion in Complex Flow Environments

Recent numerical studies have expanded from idealized uniform flows to more realistic and complex environments, revealing how external conditions can drastically alter propulsive performance. In particular, density stratification introduces buoyancy and internal waves that significantly modify the performance of the flapping hydrofoils. Two key parameters are essential to understand these effects. The first is the Brunt–Väisälä frequency (*N*), representing the natural oscillation frequency of a fluid parcel displaced vertically in a stratified medium, given by $N={\left(\gamma g/\rho_{0}\right)}^{1/2}$, where γ is the background density gradient (the density difference across the vertically stratified fluid column), *g* is the gravitational acceleration, and $\rho_{0}$ is the reference density. The second is the internal Froude number (Fr), defined as $Fr=U_{\infty }/\left(ND\right)$ (where *D* is the maximum thickness of the hydrofoil), which quantifies the ratio of inertial to buoyancy forces [80,81,82]. A low Fr indicates strong stratification, where buoyancy is dominant.

**Figure 8:**
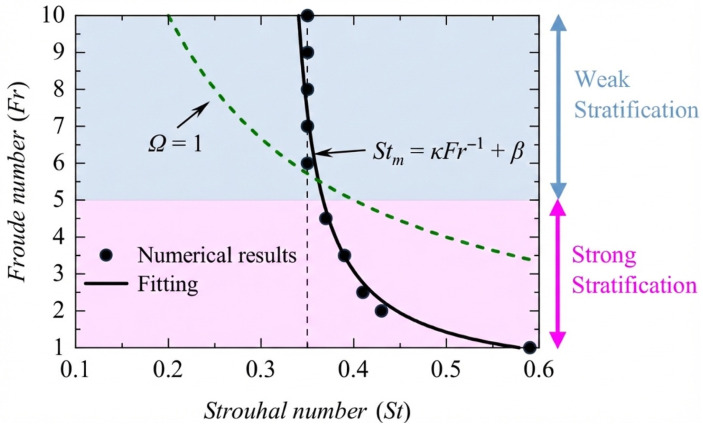
The relationship between the optimal Strouhal number ($St_{m}$) for peak propulsive efficiency and the internal Froude number (Fr). The numerical results (black dots) show that $St_{m}$ increases as stratification strengthens (Fr decreases), moving outside the typical 0.2–0.4 range for homogeneous fluids (grey dashed line at St=0.35). The black solid line shows the fit $St_{m}=\kappa Fr^{-1}+\beta$, where κ and β are fitted constants. The green dashed line indicates the boundary Ω=1 (dimensionless frequency ratio used in the source to separate regimes), with Ω<1 denoting the strongly stratified regime and Ω>1 the weakly stratified regime. Replotted from the raw data of Wang et al. [82].

For a purely pitching foil, while strong stratification (Fr=1) degrades propulsive efficiency, a moderate level of stratification (Fr=2) can lead to an 18.3% increase in efficiency compared with a homogeneous fluid, partly by stabilizing the wake structure and preventing asymmetric deflection [80]. This enhancement is even more pronounced for a combined heaving and pitching foil, where a remarkable propulsive efficiency exceeding 80% is achieved at an optimal Fr≈3.5 [81]. This high performance is linked to intense vortex interactions and the formation of a high-intensity momentum jet created by an effective wave-mean flow interaction near the foil. The optimal kinematics also shifted in stratified flow. The optimal Strouhal number for peak efficiency, typically in the 0.2–0.4 range for homogeneous flows, increases significantly in strongly stratified flows, reaching as high as St≈0.59 at Fr=1. This shift is governed by a resonance mechanism, where peak efficiency occurs when the hydrofoil flapping frequency is proportional to the fluid natural Brunt–Väisälä frequency [82]. This relationship is visualized in Figure 8, which plots the optimal Strouhal number ($St_{m}$) against the Fr. As stratification strengthens (Fr decreases), $St_{m}$ clearly increases, with the trend captured by a fitting curve (solid line) that follows the relationship $St_{m}\propto Fr^{-1}$. The underlying physical process is fluid entrainment; in strongly stratified flows, the dominant buoyancy forces confine the fluid displaced by the hydrofoil. As the flapping frequency (and thus the Strouhal number) increases, the resulting intensified density perturbations generate favorable pressure fields that enhance the thrust. This mechanism is only effective when the buoyancy is strong enough to keep the perturbed fluid close to the foil, explaining the shift to higher optimal Strouhal numbers in strongly stratified environments [82].

The concept of wave-devouring propulsion (WDP), in which a foil harnesses energy from ambient waves to generate thrust, has been extensively modeled. Both Boundary Element Methods (BEMs) and CFD approaches have been used to simulate flaps operating near a free surface and in waves, showing that these systems can effectively augment ship propulsion [25]. The numerical results indicate that the thrust coefficient can increase by as much as 20%, with the dual benefit of adding thrust and providing dynamic stabilization to the vessel [83]. The presence of a solid boundary (ground effect) has also been numerically studied. At large flapping amplitudes, the ground effect can enhance both the thrust and propulsive efficiency by altering the pressure field and vortex dynamics. However, there is a critical ground distance below which the performance rapidly degrades owing to the formation of strong adverse suction forces that pull the hydrofoil toward the ground [84].

### 4.2. Numerical Studies on Flapping Foil-Based Power Generation

In contrast to propulsion, the goal of power generation is to extract kinetic energy from fluid flow, positioning flapping foils as a potential alternative to conventional rotary turbines, particularly in low-speed or shallow-water environments. Numerical simulations are essential for optimizing the power extraction efficiency and understanding the complex interplay between foil motion and the fluid forces. Research in this area is often categorized based on the actuation mechanism of the foil. The following sections review numerical studies on idealized fully active systems to establish performance benchmarks, more practical semi-passive and fully passive systems that rely on fluid–structure interaction, advanced strategies for performance enhancement, the effects of multi-foil arrays and environmental conditions, and the development of specialized computational models.

#### 4.2.1. Parametric Studies of Fully-Active Systems

Fully active systems, in which both pitch and heave motions are prescribed, serve as idealized models for exploring the upper limits of performance. The work by Kinsey and Dumas [16] is a cornerstone in this field. Using 2D laminar simulations for a NACA0015 foil at Re=1100, they performed a comprehensive parametric study of the flapping frequency and pitch amplitude, identifying an optimal efficiency of 34% at a reduced frequency of approximately $f^{*}=0.15$ and a high pitching amplitude of $\theta_{0}\approx 75^{{}^{\circ}}$. The influence of non-sinusoidal motion profiles is also a key area of investigation. Xiao et al. [85], using an in-house Navier-Stokes solver for a NACA 0012 foil at Re = 10,000, showed that a trapezoidal-like pitching motion combined with sinusoidal heaving could significantly increase the output power over a certain Strouhal number range. Similarly, Lu et al. [86] employed the commercial code CFX to study a NACA 0012 foil at Re = 10,000 with various non-sinusoidal profiles. They concluded that the best energy extraction occurred for a tooth-like plunging profile combined with a square-like pitching profile. In their kinematic formulation, an adjustable shaping parameter *K* (e.g., $K_{\theta }$ for pitch and $K_{h}$ for plunge) continuously varies the waveform from sawtooth-like to square-like and can be interpreted as controlling how abruptly the foil reverses direction.

**Figure 9:**
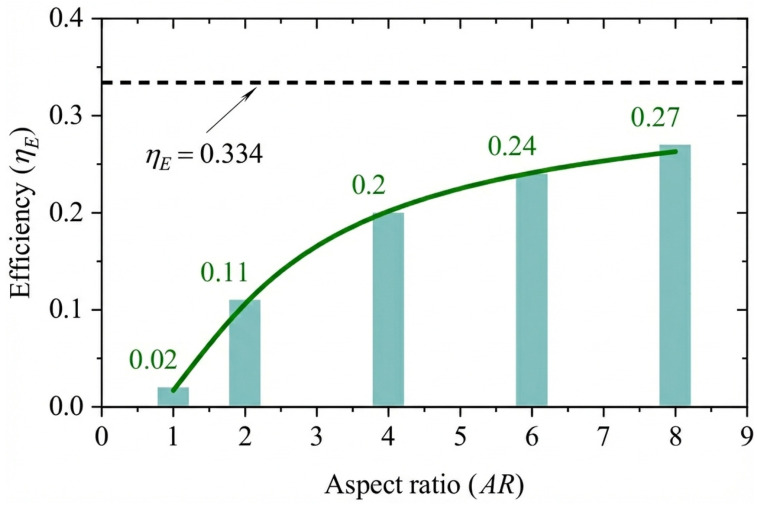
Variation of energy extraction efficiency ($\eta_{E}$) as a function of AR. The squares represent the simulation data points, while the solid line indicates the empirical fit $\eta_{E}=0.334/{\left[1+1/\left(AR-0.75\right)\right]}^{1.85}$. The dashed horizontal line represents the efficiency of the equivalent two-dimensional flow ($\eta_{E}=0.334$). Replotted from the raw data of Deng et al. [87].

Numerical simulations conducted by Deng et al. [87] using OpenFOAM at Re=1100 revealed significant performance degradation owing to 3D effects. This study highlighted a qualitative transition in wake topology for low aspect ratios, where the intensity of the leading-edge vortices (LEVs) was weakened by end effects extending approximately 1.2c from the foil tips. As illustrated in Figure 9, the authors identified a critical aspect ratio of AR=4 for sinusoidal motions. Below this threshold, 3D flow characteristics dominate, whereas increasing the aspect ratio beyond AR=4 yields diminishing hydrodynamic returns that may be offset by the structural costs associated with high-aspect-ratio foils. Recent advancements in computational methods have introduced data-driven approaches to overcome the high computational costs of traditional CFD in fully-active systems. Li et al. [88] developed a real-time energy extraction model using a Convolutional Neural Network (CNN) framework containing two modular networks: one for predicting transient physical fields (pressure and velocity) and another for aerodynamic characteristics (the lift and pitching-moment coefficients, $C_{L}$ and $C_{M}$). Their deep learning model, trained on numerical simulation datasets, could predict flow fields and efficiency in milliseconds. Furthermore, by leveraging the automatic differentiation mechanism inherent in deep learning, they performed gradient-based optimization to identify optimal kinematic parameters ($f^{*},\theta_{0},h_{0}$) in a fraction of the time required by conventional solvers. Similarly, addressing the need for efficient time-series prediction, Saeed et al. [89] proposed a Reduced-Order Model (ROM) combining Proper Orthogonal Decomposition (POD) with Long Short-Term Memory (LSTM) neural networks. Simulating a NACA 0012 foil at Re=1100, they utilized the LSTM network to predict the temporal coefficients of pressure POD modes. This approach allowed for the accurate reconstruction of hydrodynamic forces and power generation capacity for long time durations beyond the training interval, offering a robust alternative to traditional Galerkin projection-based ROMs.

#### 4.2.2. Semi-Passive and Fully-Passive Systems

**Figure 10:**
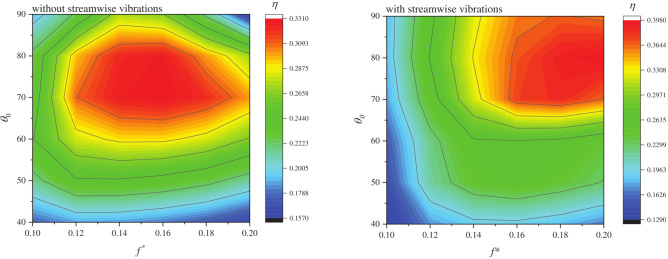
Energy extraction efficiency ($\eta_{E}$; denoted as η in Bai and Zheng [93]) contours in the parameter space of reduced frequency ($f^{*}$) and pitching amplitude ($\theta_{0}$) for the 1-DOF system without streamwise vibrations (**left**) and the 2-DOF system with streamwise vibrations (**right**). The 2-DOF configuration shifts the optimal efficiency region to higher $f^{*}$ and $\theta_{0}$. Reprinted with permission from Bai and Zheng [93].

To simplify mechanical complexity, semi-passive (one motion prescribed, one induced) and fully passive (both motions induced) systems are of great practical significance. Zhu and Peng [37] modeled a semi-passive system with prescribed pitching using a Navier-Stokes solver for a Joukowsky foil at $Re\approx 10^{3}$, finding that positive net energy extraction was only possible at low frequencies and was highly dependent on the pivot axis location. Deng et al. [90] used OpenFOAM at Re=1000 to study the inertial effects in a semi-passive system with prescribed pitching ($\theta_{0}=75^{{}^{\circ}}$, $f^{*}=0.16$), identifying that the energy harvesting efficiency decreases monotonically with increasing mass ratio. Teng et al. [91], also using OpenFOAM for a semi-passive NACA 0015 foil at Re=1000, studied pitching amplitudes of $\theta_{0}=45^{{}^{\circ}}$ and $75^{{}^{\circ}}$ and found that the benefits of non-sinusoidal pitching were limited to cases with small pitching amplitudes. A different semi-passive configuration with prescribed heave and passive pitch was numerically investigated by Boudreau et al. [18] using RANS simulations at a high Re of $3.9\times 10^{6}$ and a pivot axis at the quarter chord. They achieved a remarkable efficiency of 45.4%, demonstrating the high potential of this simpler concept. The effect of Re on a semi-passive system ($f^{*}=0.1$ and $\theta_{0}=15^{{}^{\circ}}$, with other setup parameters as defined in the source) was systematically studied by Javed et al. [92] using a hybrid meshfree-Cartesian grid method for an NACA 0015 foil at Re = 5000–50,000. They observed that the net power extracted increased at higher Re. More recently, Bai and Zheng [93] utilized Fluent to investigate an active pitching NACA 0015 foil at Re=1100 subject to two-degree-of-freedom (2-DOF) vortex-induced vibrations (VIV). Their results, presented in Figure 10, revealed that releasing the streamwise DOF fundamentally modified the energy harvesting response. The 2-DOF system harnesses drag forces, a mechanism unavailable in the traditional 1-DOF constrained case, which shifts the optimal operating regime toward higher reduced frequencies ($f^{*}\approx 0.16–0.20$) and larger pitching amplitudes ($\theta_{0}\approx 85^{{}^{\circ}}$). Consequently, this configuration enhances the maximum efficiency by approximately 20% relative to that of a traditional system, achieving peak efficiencies nearing 40%.

For fully passive systems, Young et al. [20] conducted a 2D Navier–Stokes FSI simulation of a NACA0012 foil at Re=1100 and $1.1\times 10^{6}$. They investigated both pitch-angle and angle-of-attack control methodologies, achieving a high efficiency of 41% with nonsinusoidal angle-of-attack control, demonstrating the importance of managing LEV dynamics. Wang et al. [94] used an Immersed Boundary (IB) method at Re=400 to study a fully passive NACA 0012 foil, identifying five distinct response regimes and showing that the highest efficiency of 32% occurred in a stable synchronization regime characterized by harmonic wake-body interaction.

#### 4.2.3. Flow Control and Performance Enhancement

Active flow control techniques have been numerically applied to further improve efficiency. Wu et al. [95] used an immersed boundary-lattice Boltzmann method (IB-LBM) for a semi-active elliptic airfoil at Re=1100 and demonstrated that a pair of synthetic jets on the foil’s surface could enhance energy harvesting efficiency by increasing the lift force when operated with suitable parameters. Hoke et al. [96] used a Navier-Stokes solver to investigate active morphing of a NACA 0015 foil (Re=1100) and a flat plate (Re = 10,000). By morphing the leading edge (LE), trailing edge (TE), or both, they achieved efficiency gains of up to 29.7% for the NACA 0015 foil and 36.2% for the flat plate over their rigid baselines. They identified three physical mechanisms responsible for this improvement: changes in the projected area, variation in LEV shedding timing, and proximity of the shed vortex to the foil.

**Figure 11:**
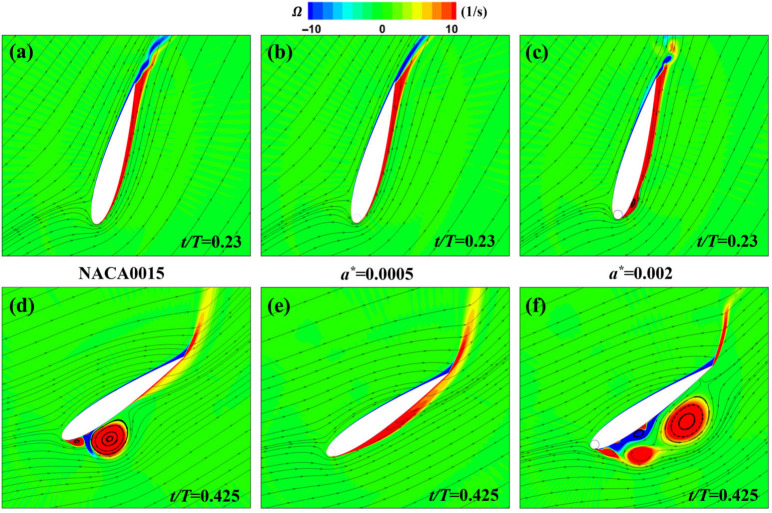
Instantaneous vorticity contours and streamlines at t=0.23T (**top**) and t=0.425T (**bottom**), where *T* is the oscillation period. The nondimensional gap width is $a^{*}=a/c$, where *a* is the cylinder–foil gap. (**a**,**d**) The baseline NACA 0015 exhibits pronounced LEV formation and separation. (**b**,**e**) The Magnus Effect Flapping Wing (MEFW) configuration with $a^{*}=0.0005$ maintains attached flow by energizing the boundary layer. (**c**,**f**) A wider gap ($a^{*}=0.002$) fails to suppress separation due to reverse leakage flow through the gap. Reprinted from Zhang et al. [97].

A composite design was investigated by Zhang et al. [97], who numerically simulated a flapping NACA 0015 foil at $Re=5\times 10^{5}$ with a leading-edge rotating cylinder. They termed this configuration a Magnus Effect Flapping Wing (MEFW). The study optimized the gap width $a^{*}$, the rotation speed ratio *R* (cylinder surface speed normalized by $U_{\infty }$), and the phase $\phi_{0}$ (phase offset between cylinder rotation and the flapping cycle). They reported that a small gap ($a^{*}=0.0005$) is critical for limiting reverse leakage flow that can trigger separation. As shown in Figure 11, the baseline foil and wider-gap cases ($a^{*}=0.002$) exhibit large-scale separation, whereas the optimized MEFW ($a^{*}=0.0005,\;R=3$) maintains attachment and improves energy harvesting performance.

#### 4.2.4. Multi-Foil Configurations and Environmental Effects

The arrangement of multiple foils and the influence of the surrounding environment are critical for their practical applications. Wang et al. [98] numerically studied two parallel NACA 0012 foils at Re=1100 using a laminar solver in Fluent. They found that the interaction between the foils affected the performance and that non-sinusoidal motion could further enhance power extraction. For tandem configurations, Ma et al. [99] used Fluent at $Re\approx 10^{6}$ to analyze two fully passive hydrofoils coupled by a hydraulic system. They observed that the upstream foil exhibited a higher heaving amplitude and performance. A key insight was that, unlike prescribed-motion systems, this passive configuration naturally adjusted its response to weaken and avoid the strong, potentially destructive or beneficial wake interactions that are the focus of active systems.

Environmental effects, such as ground proximity, free surfaces, and unsteady inflow, have also been modeled. Wu et al. [100], using an IB-LBM for a semi-active foil at Re=1100, found that the presence of one or two solid walls (ground effect) could significantly improve net power extraction efficiency with improvements of up to 211% for two walls. They attributed this mainly to an increase in the lift force. He et al. [101] confirmed this using OpenFOAM for a semi-passive NACA 0015 foil at Re=500,000, showing a 17.77% power improvement at an optimal ground clearance. They attributed this to a Venturi-like effect in the foil–ground gap, where the reduced clearance accelerates the local flow and lowers pressure, which increases circulation and strengthens near-foil vortices. This strengthened vortex field interacts more effectively with the foil motion, improving the force–motion phase relationship and thereby increasing net extracted power. By contrast, the free surface was found to be detrimental. Deng et al. [102] studied a fully active NACA 0015 foil at Re=900 using STAR-CCM+ with a Volume of Fluid (VOF) method, concluding that the free surface played an unfavorable role by reducing the power extracted from heaving motion while increasing the power input required for pitching. For unsteady inflow, Zhan et al. [103] used Fluent to simulate a semi-active NACA0015 foil in gusty flow (Re=1100) and found that a stronger gust fluctuation amplitude generated a higher power extraction efficiency. The effect of stratified environments was explored by Wang et al. [104] for a fully activated flapping foil at Re=1000. Their results indicated that density stratification generally decreases energy harvesting efficiency due to poor force-motion synchronization. However, a notable exception was found at a strong stratification level (Fr=1), where a sudden increase in the extracted power occurred, which was attributed to the distinct flow structures generated by the induced internal waves.

In short, numerical simulations have enabled systematic exploration of kinematic, geometric, and environmental effects, and they provide access to flow structures that are difficult to resolve experimentally. In addition, the strongest gains often depend on modelling choices such as dimensionality, turbulence treatment, and boundary conditions, which can affect quantitative predictions. This motivates the following discussion of current limitations and future directions, including validation needs, robustness in realistic environments, and scalable design rules.

## 5. Challenges, Limitations and Future Outlook

Despite significant progress in exploring the hydrodynamics of flapping foils, a dichotomy remains between the idealized environments of computational models and the complex realities of physical deployment. The following section delves into these challenges, highlighting the specific limitations identified in the literature and outlining potential paths forward.

### 5.1. Numerical Challenges and Computational Outlook

#### 5.1.1. The Gap in Fidelity: From 2D RANS to 3D LES/DNS

A predominant limitation of current CFD is the reliance on 2D URANS simulations. While 2D models, such as those used by Ren et al. [62] using DVM or Alberti et al. [61], successfully capture fundamental thrust mechanisms such as the Knoller-Betz effect, they inherently fail to predict finite-span phenomena. In the context of propulsion, Lagopoulos et al. [105] demonstrated that 2D simulations neglect critical 3D vortex dynamics, specifically the formation of tip vortices that interact destructively with the spanwise LEV. Similar 3D vortex breakdown mechanisms have been observed in energy extraction studies by Mo et al. [106], confirming that 2D assumptions fundamentally misrepresent the wake topologies in finite-span applications. Furthermore, Dong et al. [65] established that low AR foils exhibit interconnected vortex loops and rings, a topology fundamentally different from the inverse von Kármán streets assumed in infinite-span models. Therefore, high-fidelity 3D LES/DNS are required to accurately capture vortex interconnectivity. Future numerical work could focus on the “force partitioning” of these 3D structures to isolate lift and drag contributions, as explored phenomenologically by Raut et al. [79].

#### 5.1.2. Modeling Complex Environmental Interactions

Numerical models often assume homogeneous, infinite fluid domains, neglecting the environmental stratification and boundaries found in the oceans. Recent studies have shown that fluid density significantly alters the wake dynamics and propulsive efficiency [80,81,82], yet this is rarely considered in standard AUV design codes. Furthermore, accurately coupling free-surface wave deformation with hydrofoil hydrodynamics remains a challenge at low submergence depths. Filippas and Belibassakis [83] noted the necessity of linearizing free-surface conditions in BEM to manage computational cost, which limits accuracy for breaking waves. Similarly, He et al. [101] highlighted the complexity of resolving these interactions in power extraction scenarios involving wing-in-ground effects. Advanced solvers must integrate multiphase flows to resolve free surface conditions and stratification to accurately predict propulsion in realistic sea environments.

#### 5.1.3. AI-Driven Optimization and Modeling

Traditional parametric sweeps in flapping foil-based studies are often computationally expensive and limited to predefined kinematic patterns (e.g., sinusoidal motion). As noted by Bao et al. [73], human-designed trajectories may not be optimal in unsteady flow regimes. Bai and Zheng [93] also highlight the complexity of optimizing 2-DOF VIV systems for energy harvesting, where coupling effects are highly nonlinear. The integration of DRL represents a critical frontier for addressing these nonlinearities and computational bottlenecks. Recent studies have moved beyond simple parametric optimization. Wang et al. [74] demonstrated that DRL agents utilizing Transformer architectures can autonomously discover effective non-parametric flapping paths, significantly outperforming sinusoidal baselines by manipulating vortex shedding timing. Furthermore, to mitigate the high cost of high-fidelity CFD, deep learning is increasingly being used to construct real-time surrogate models. Li et al. [88] and Saeed et al. [89] successfully developed CNN and LSTM-based frameworks, respectively, to predict unsteady flow fields and aerodynamic loads in milliseconds. These advancements suggest that the future of computational research lies in the development of “Digital Twins” hybrid frameworks where deep learning surrogates provide real-time feedback for optimization and control, with high-fidelity CFD used sparsely for training and validation.

### 5.2. Experimental Challenges and Physical Viability

#### 5.2.1. Mechanics of Fluid–Structure Coupling and Flexibility

A major challenge in current experiments is to make flexible foils move in sync with water flow without active control. Sharma and Dutta [48] showed that flexibility along the chord of the foil can improve propulsion by delaying drag and increasing thrust. However, applying these benefits to energy harvesting is difficult. Totpal et al. [55] found that when a foil deforms passively due to its own weight and motion (inertia), it often fails to match the timing of the peak power generation phase. Consequently, this timing mismatch can result in flexible foils producing less power than rigid foils. Therefore, future experiments could move beyond simple flexible materials toward mechanisms with “tunable” stiffness. New setups should utilize smart materials or adjustable springs to actively control the deformation of the foil, ensuring that the benefits observed in propulsion can also be applied to energy generation.

#### 5.2.2. Energy Costs of Activation

For semi-active systems, the energy required to drive the motion, which is usually pitching, is a critical problem that is often ignored in simulations. Jiacheng et al. [54] found that the power consumed by the pitching mechanism can cancel out the energy harvested, especially when the pitching period is short. Similarly, Wang et al. [49] noted that although waves provide “free” energy for heaving, the tuning of the pitch spring stiffness is highly sensitive. If the stiffness is not optimal, the foil does not move in phase with the wave, which drastically reduces the efficiency. Future experimental designs should focus on reducing mechanical losses. This could include developing drive systems that recover energy during the motion cycle or exploring fully passive systems that do not require motors.

#### 5.2.3. Operational Environment and Three-Dimensionality

Early fundamental studies, such as those by Schouveiler et al. [42] and Schnipper et al. [107], were mostly conducted under steady, uniform water flows. However, real-world applications must deal with random waves and complex boundaries that are difficult to replicate in testing tanks. Belibassakis et al. [51] successfully demonstrated that foils can improve ship propulsion in a towing tank, but they noted that scaling these results to irregular sea states is difficult because ship motions (heave and pitch) become random. Furthermore, Quinn et al. [50] showed that the time-averaged thrust increases as the foil approaches a solid boundary and that the mean lift can change sign over different clearance ranges, indicating sensitivity to wall distance. These results imply that the performance benefit depends on maintaining an appropriate clearance. In practical settings with unsteady waves and uneven seabeds, particularly in nearshore or shallow-water deployments, clearance control and conservative operating margins are therefore important to avoid adverse loading and unintended contact.

Finally, standard experiments often use end plates to approximate 2D flow, which hides complex 3D wake instabilities. Future diagnostics should prioritize the broader application of volumetric measurements, such as Tomographic PIV. Although these techniques have effectively revealed complex 3D flow patterns in aerodynamic flapping wings [108,109], they are rarely applied to flapping foil-based hydrodynamic propulsion and power generation applications. Increasing their use would allow for a complete observation of 3D wake structure breakdown in finite-span foils, thereby connecting simplified 2D experiments with real-world 3D conditions.

## 6. Conclusions

This review provides a comprehensive synthesis of the state of the art in flapping foil technology, bridging the domains of bio-inspired propulsion and power generation. By unifying these fields under a shared hydrodynamic framework, this review clarifies the operational duality of oscillating foils. Classical theory utilizes the feathering parameter to theoretically delimit the transition between these modes, where low values indicate momentum transfer for propulsion, and high values enable kinetic energy extraction. However, this review demonstrates that maximizing performance within these regimes requires moving beyond single-parameter metrics to the multi-dimensional optimization of the Strouhal number, reduced frequency, and phase synchronization.

A critical examination of the experimental foundations reveals that although optimal propulsion in homogeneous fluids is consistently associated with a narrow range of Strouhal numbers, typically between 0.2 and 0.4, real-world deployment requires a departure from these idealized baselines. Experimental evidence confirms that incorporating passive structural flexibility, specifically chordwise deformation, significantly enhances thrust and stabilizes wake structures. However, the application of flexibility to power generation remains a challenge. Passive deformation often fails to synchronize with the peak power production phase, suggesting that future harvesting systems require tunable stiffness mechanisms or active control to match unsteady flow physics.

A distinguishing feature of this review is the detailed analysis of flapping foils in complex, non-homogeneous environments, a domain often overlooked in standard design codes. Numerical investigations have highlighted that density stratification fundamentally alters the hydrodynamic landscape. In strongly stratified flows characterized by low Fr, the optimal flapping frequency shifts significantly because of resonant interactions with the natural Brunt–Väisälä frequency of the fluid. This finding indicates that future bio-inspired AUVs operating in the ocean thermocline could significantly enhance propulsive efficiency by tuning kinematics to exploit fluid-density resonance and confinement effects rather than relying on homogeneous flow assumptions.

A review of computational methodologies indicates a paradigm shift from traditional parametric sweeps to high-fidelity and data-driven approaches. Although 2D simulations have established fundamental scaling laws, they often fail to capture the critical finite-span vortex interconnectivities inherent to low-aspect-ratio foils. Consequently, the field is moving toward 3D LES and the integration of DRL. These AI-driven frameworks can autonomously discover non-parametric, high-efficiency trajectories that outperform human-designed sinusoidal motions, offering a pathway to digital twin systems capable of real-time flow adaptation.

The advancement of flapping foil technology hinges on resolving the dichotomy between computational idealization and physical reality. Future research must prioritize three key areas: the transition from rigid to tunable stiffness materials that can adaptively modulate deformation, the development of high-fidelity coupled solvers that accurately resolve multiphase free-surface effects and stratification, and the experimental validation of AI-optimized trajectories in chaotic turbulent flow environments. By addressing these challenges, flapping foil systems can evolve from laboratory concepts to viable, high-efficiency solutions for next-generation marine robotics and sustainable power generation.

## Data Availability

Data are contained within the article.

## Abbreviations

The following abbreviations are used in this manuscript:

LES	Large-Eddy Simulation
DRL	Deep Reinforcement Learning
AUV	Autonomous Underwater Vehicle
LEV	Leading-Edge Vortex
FSI	Fluid–Structure Interaction
CFD	Computational Fluid Dynamics
WDP	Wave-Devouring Propulsion
NACA	National Advisory Committee for Aeronautics
LDV	Laser Doppler Velocimetry
PIV	Particle Image Velocimetry
OFEC	Oscillating Foil Energy Converter
FAPG	Flapping Airfoil Power Generator
MAV	Micro Aerial Vehicle
URANS	Unsteady Reynolds-averaged Navier–Stokes
DG	Discontinuous Galerkin
RANS	Reynolds-averaged Navier–Stokes
DVM	Discrete Vortex Method
CST	Class-Shape Transformation
ALE	Arbitrary Lagrangian–Eulerian
TEV	Trailing-Edge Vortex
PPO	Proximal Policy Optimization
DNS	Direct Numerical Simulation
WAP	Wave-assisted Propulsor
ROM	Reduced-Order Model
POD	Proper Orthogonal Decomposition
LSTM	Long Short-Term Memory
DOF	Degree-of-Freedom
VIV	Vortex–Induced Vibration
IB-LBM	Immersed Boundary–Lattice Boltzmann Method
MEFW	Magnus Effect Flapping Wing
VOF	Volume of Fluid
